# QRS Index as a Predictor of Response to Cardiac Resynchronization Therapy: A Systematic Review and Meta-Analysis

**DOI:** 10.3390/jcm15083074

**Published:** 2026-04-17

**Authors:** Egle Corrado, Francesco Stabile, Sebastian Jaramillo, Mariana Niño Lopez, Marco Mirabella, Cristina Madaudo, Vincenzo Sucato, Alfredo Ruggero Galassi, Roberto De Ponti, Giuseppe Coppola

**Affiliations:** 1 Division of Cardiology, Department of ProMISE, University Hospital P. Giaccone, University of Palermo, 90127 Palermo, Italy; egle.corrado@unipa.it (E.C.); marco.mirabella@live.it (M.M.); vincenzo.sucato@unipa.it (V.S.); giuseppe.coppola@policlinico.pa.it (G.C.); 2 Facultad Ciencias Biomédicas, Universidad Austral, Av. Juan Domingo Peron, 1500-Pilar, Buenos Aires B1629, Argentina; sebastianjaramro@gmail.com; 3 Beth Israel Deaconess Medical Center, Boston, MA 02215, USA; ninolopezmariana@gmail.com; 4 Department of Medicine and Surgery, University of Insubria, 21100 Varese, Italy; roberto.deponti@uninsubria.it

**Keywords:** cardiac resynchronization therapy, QRS index, heart failure, CRT response

## Abstract

**Background**: Cardiac resynchronization therapy (CRT) improves outcomes in heart failure (HF) patients with reduced left ventricular ejection fraction (LVEF) and a wide QRS complex. However, up to 30–50% of patients fail to respond. The QRS Index, which quantifies QRS shortening after CRT, has emerged as a potential predictor of response. We aimed to perform a systematic review and meta-analysis to evaluate the association between QRS Index and CRT response. **Methods**: We searched PubMed, Scopus and Cochrane for studies reporting QRS Index values in CRT responders and non-responders. Studies defining response based on clinical, echocardiographic, or combined criteria were included. Heterogeneity was assessed using the I^2^ statistic, and a random-effects model was applied. A meta-regression analysis explored the relationship between baseline echocardiographic parameters and QRS Index. **Results**: Nine studies with 1274 patients met the inclusion criteria, with 760 (59%) classified as responders and 514 (41%) as non-responders. The weighted mean ± standard deviation was 16.14 ± 13.19 in responders and 7.22 ± 14.96 in non-responders. The QRS Index was significantly higher in the responder group compared to non-responders (mean difference: 8.76; 95% CI: 6.45–11.06; I^2^ = 45%; *p* < 0.00001). Meta-regression revealed that lower left ventricular end-systolic volume (LVESV) values were associated with even higher QRS Index in responders compared to non-responders (β = −0.0483; 95% CI: −0.0938; −0.0029, *p* = 0.0372). **Conclusions**: QRS Index is significantly higher in CRT responders, supporting its role as a predictor of response. Further studies are needed to standardize its clinical use and assess its prognostic impact.

## 1. Introduction

Cardiac resynchronization therapy (CRT) is a treatment for heart failure (HF) patients with reduced left ventricular ejection fraction (LVEF) and a wide QRS complex. It aims to improve survival rates, decrease HF symptoms, and reduce hospitalizations [1]. QRS duration is part of the eligibility criteria for recommending CRT implantation, being a powerful predictor of the effects of CRT on morbidity and mortality in patients with symptomatic HF and left ventricular systolic dysfunction who are in sinus rhythm [2]. Despite current selection criteria, up to 30–50% of patients who received CRT did not experience any beneficial results [3,4,5]. Consequently, there has been growing interest in identifying variables that predict response to CRT during or shortly after device implantation to optimize patient management. Some authors have found that baseline ECG is of limited prognostic usefulness and that QRS shortening during CRT may be a better marker of outcome than baseline QRS duration [6]. Nevertheless, the role of QRS shortening during CRT as a predictor of positive response to CRT remains controversial. Previous meta-analyses have shown that a greater reduction in QRS width is associated with clinical and echocardiographic response to CRT [7,8]. In this context, the QRS Index, calculated as [(native QRS duration—QRS duration during CRT)/native QRS duration × 100], emerges as an effective and easy-to-measure parameter for predicting response to CRT, as it quantifies the extent of QRS shortening after CRT implantation relative to baseline QRS duration [9,10]. Different studies have investigated the predictive role of this parameter; however, due to the small sample size, they lacked sufficient statistical power to assess its independent prognostic value. Therefore, we aimed to perform a systematic review and meta-analysis to evaluate and compare QRS Index values between CRT responders and non-responders.

## 2. Methods

This systematic review and meta-analysis were performed and reported in accordance with the Cochrane Collaboration Handbook for Systematic Review of Interventions and the Preferred Reporting Items for Systematic Reviews and Meta-Analysis (PRISMA) Statement guidelines [11,12] (Table S1, Supplementary Materials).

The prospective meta-analysis protocol was registered on PROSPERO on 26 January 2025, under protocol number CRD42025642270.

Studies included in this meta-analysis met all the following eligibility criteria: (1) prospective or retrospective observational studies; (2) enrollment of patients eligible for CRT implantation according to established guideline criteria; (3) availability of QRS Index or of an equivalent parameter; and (4) direct comparison between responders and non-responders to CRT based on clearly defined response criteria, including echocardiographic response (typically defined as ≥10–15% reduction in LVESV or improvement in LVEF), clinical response (improvement in NYHA functional class), or combined clinical-echocardiographic definitions. A detailed summary of response definitions adopted in each study is provided in Supplementary Table S2.

Studies were excluded if they: (1) did not provide a clear definition of response to CRT; (2) did not include a comparative analysis between responders and non-responders; (3) did not report QRS Index or an equivalent parameter; or (4) contained overlapping patient populations with other included studies. To standardize differences across studies, the QRS Index was considered positive in patients who experienced a reduction in QRS duration and negative in those with an increase in QRS duration following CRT. The primary endpoint was the difference in QRS Index between responders and non-responders to CRT.

### 2.1. Search Strategy and Data Extraction

We systematically searched PubMed, Scopus and Cochrane Central Register of Controlled Trials from their inception to 19 January 2025 with the following search strategy: (“cardiac resynchronization” OR CRT) AND (“QRS index” OR “QRS width” OR “QRS change” OR “QRS narrowing” OR “QRS shortening” OR “QRS duration”) AND (response OR responders).

The references from all included studies, previous systematic reviews and meta-analyses were also manually searched for any additional studies.

Two authors (F.S. and M.M.) independently performed the search and extracted the data according to pre-defined search criteria. Any disagreements were resolved by consensus between the authors.

### 2.2. Quality Assessment

We evaluated the risk of bias in observational studies using the Risk Of Bias In Non-randomized Studies—of Exposures (ROBINS-E Version 24 March 2024) [13]. Two independent authors completed the risk of bias assessment (F.S. and S.J.). Disagreements were resolved through consensus after discussing reasons for discrepancies. Publication bias was investigated by funnel-plot analysis of point estimates in relation to study weights.

### 2.3. Statistical Analysis

Mean differences (MDs) with 95% confidence intervals (CIs) were used to compare the QRS Index between responders and non-responders. We assessed heterogeneity with the Cochran Q test and I^2^, with <25% indicating low, 25% to 50% indicating low to moderate, 50% to 75% indicating moderate to high and >75% indicating high heterogeneity. We adopted the Restricted Maximum Likelihood (REML) random-effects model.

The QRS Index in individual studies was reported as means and standard deviations (SD). In studies that provided medians and interquartile ranges (IQRs), these values were converted into means and SDs using established statistical methods [14,15]. One study did not report the SD, which was therefore imputed from the SDs of the other studies included.

A box plot was used to graphically represent the differences in QRS Index between responders and non-responders to CRT.

A subgroup analysis was conducted, stratifying studies based on the predefined outcome measures: those assessing both clinical and echocardiographic response versus those evaluating echocardiographic response alone. Additionally, a leave-one-out sensitivity analysis was performed to ensure the robustness of the results.

A meta-regression analysis, with graphical representation using a bubble plot, was performed to assess the correlation between baseline variables (LVEF, LVESV, LVEDV, and QRS duration) and the difference in QRS Index between responders and non-responders.

We used Cochrane RevMan (Version: 8.13) for the generation of the forest plot. R (Version 2024.12.0.467) was utilized for the graphical representation of the box plot and for the meta-regression analysis. *p*-values of less than 0.05 were considered statistically significant.

## 3. Results

### 3.1. Study Selection and Baseline Characteristics

**Figure 1:**
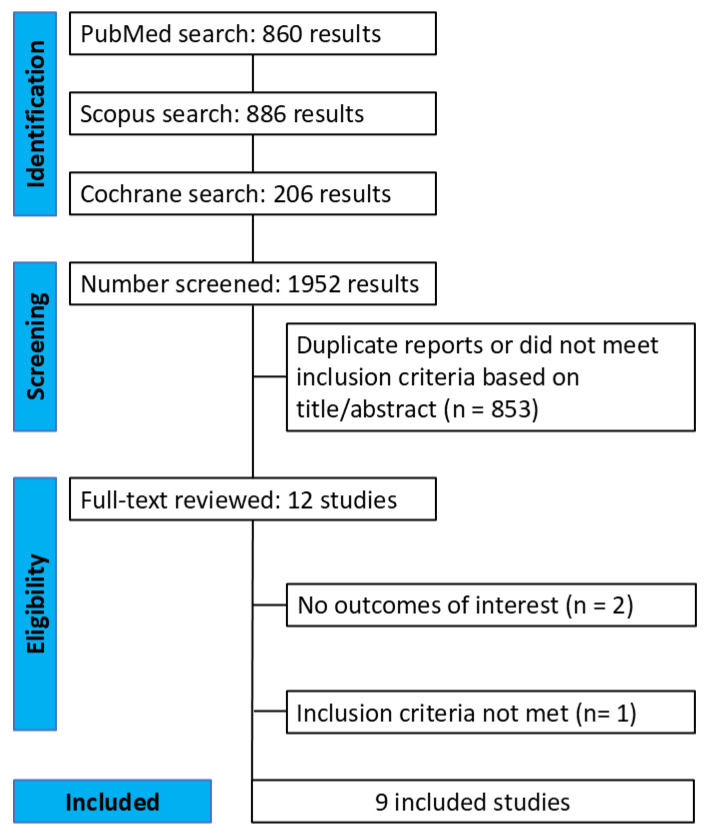
PRISMA flow diagram of study screening and selection.

The search strategy yielded 1952 results (Figure 1). After the removal of duplicate records and studies with an exclusion criterion based on title and abstract review, 12 remained and were fully reviewed for the inclusion and exclusion criteria. Nine observational studies met all inclusion criteria. A total of 1274 patients who had undergone CRT were included, with 760 (59%) classified as responders and 514 (41%) as non-responders. The baseline characteristics of the study population are presented in Table 1.

### 3.2. Quality Assessment

The quality of the studies was assessed using ROBINS-E, which showed a moderate overall risk of bias across all included studies. A detailed domain-level assessment (including confounding bias, selection bias, exposure measurement bias, outcome assessment bias, and reporting bias) is reported in Supplementary Table S5.

A funnel-plot analysis was conducted to assess the presence of publication bias. The plot showed a relatively symmetric distribution of studies around the pooled effect estimate, suggesting a low likelihood of publication bias (Figure S4).

### 3.3. Pooled Analysis

**Figure 2:**
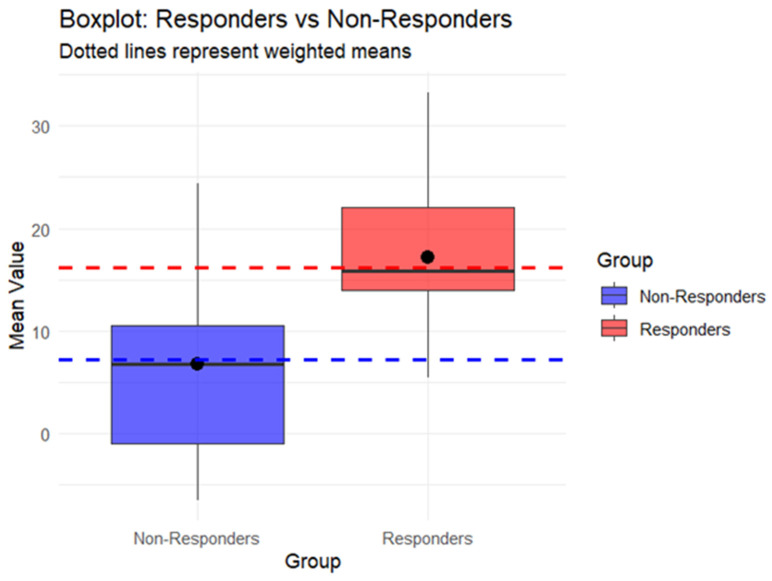
Box plot showing median (center line), mean (black dot), weighted mean (dotted lines) and IQR (box) of QRS Index between CRT responders and non-responders.

**Figure 3:**
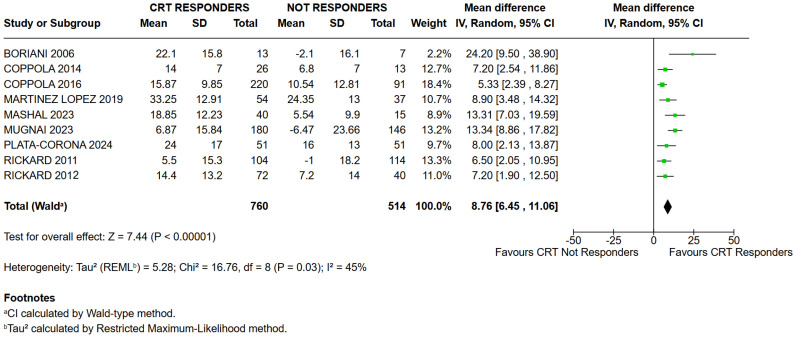
QRS Index was significantly higher in CRT responders compared to non-responders [9,10,16,17,18,19,20,21,22,23].

The median QRS Index was 15.87 in the responder group and 6.80 in the non-responder group. The weighted mean ± standard deviation, adjusted for the sample size of individual studies, was 16.14 ± 13.19 in responders and 7.22 ± 14.96 in non-responders (Figure 2). The overall analysis revealed a significantly higher QRS Index in the responder group compared to non-responders (Mean Difference: 8.76; 95% CI: 6.45–11.06; I^2^ = 45%; *p* < 0.00001; Figure 3).

### 3.4. Sensitivity Analysis

**Figure 4:**
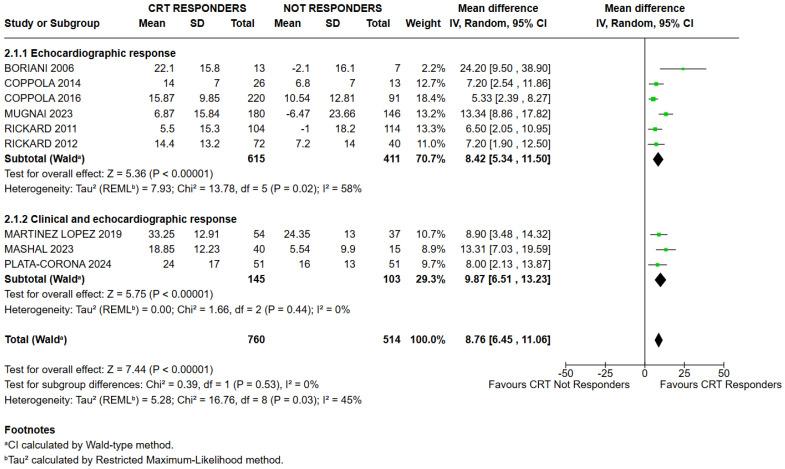
The subgroup analysis, based on the criteria used to define response to CRT, yielded results consistent with the primary analysis [9,10,16,17,18,19,20,21,22,23].

The subgroup analysis, based on the definition of response to CRT adopted in each study, yielded results consistent with the primary analysis. The test for subgroup differences (*p* = 0.53; I^2^ = 0%) did not reveal statistically significant differences between studies that defined responders using both clinical and echocardiographic criteria (Mean Difference: 9.87; 95% CI: 6.51–13.23; I^2^ = 0; *p* < 0.00001) and studies that assessed response based on echocardiographic criteria alone (Mean Difference: 8.42; 95% CI: 5.34–11.50; I^2^ = 58%; *p* < 0.00001; Figure 4). These findings confirm that QRS Index values were significantly higher in CRT responders than in non-responders, regardless of the response definition applied.

To assess the robustness of our findings and ensure that no single study disproportionately influenced the overall results, we conducted a leave-one-out sensitivity analysis. We systematically removed one study at a time from the meta-analysis and recalculated the pooled estimates. The analysis showed that the effect sizes remained stable, confirming the robustness of our findings and indicating that no single study disproportionately influenced the overall results (Table S3, Supplementary Materials).

**Figure 5:**
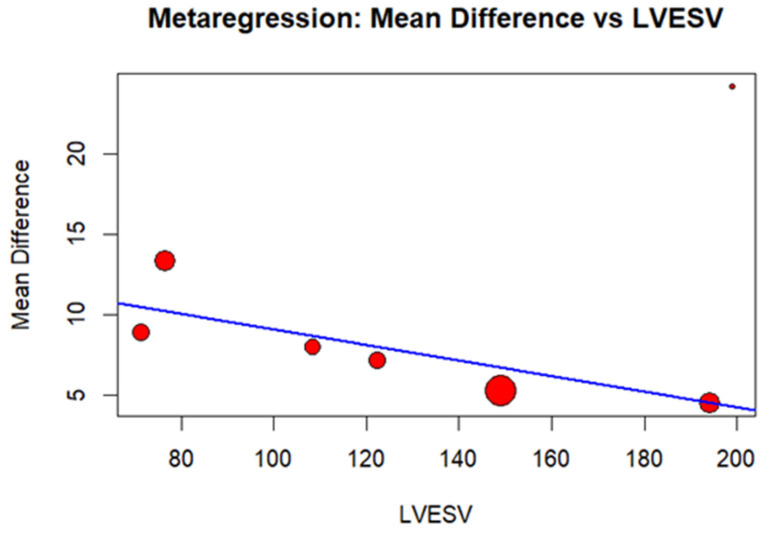
Bubble plot showing relationship between LVESV and difference in QRS Index between CRT responders and non-responders. LVESV: Left ventricular end-systolic volume. The size of the bubbles represents the weight of each study, calculated according to a random-effects model. Blue line: Linear prediction.

The meta-regression analysis of LVEF, LVEDV, LVESV, and QRS duration identified a slight inverse association between baseline LVESV and QRS Index, indicating that with lower LVESV values, the QRS Index shows even higher increases in CRT responders compared to non-responders (β = −0.0483; 95% CI: −0.0938–−0.0029, *p* = 0.0372; Figure 5 and Table S4, Supplementary Materials). No correlations were found with baseline LVEF, LVEDV, and QRS duration (Table S4, Supplementary Materials and Figures S1–S3).

## 4. Discussion

In this systematic review and meta-analysis of 9 studies and 1274 patients, we compared QRS Index values between CRT responders and non-responders, as defined by clinical and echocardiographic criteria. The results of our study highlight that QRS Index values were higher in CRT responders compared to non-responders. A greater shortening of QRS width was associated with a higher incidence of clinical and echocardiographic response.

From a clinical perspective, although pooled threshold values could not be derived due to a lack of individual patient data, available studies suggest that responders tend to show QRS Index values approximately two-fold higher than non-responders. In our pooled analysis, the mean QRS Index was approximately 16% in responders compared with approximately 7% in non-responders. These findings suggest that greater QRS shortening may identify patients more likely to benefit from CRT, although prospective studies are required to establish clinically actionable cut-off values.

Several studies have demonstrated that baseline QRS duration alone poorly correlates with clinical and echocardiographic outcomes [24]. Consequently, the QRS Index was introduced to enhance predictive accuracy by adjusting the absolute reduction in QRS duration after CRT for baseline QRS duration [9]. More prolonged QRS duration indicates more severe electrical disease as it strongly correlates with the degree of delayed left-ventricular regional activation with left bundle branch block or during right ventricular pacing [25]. Assuming that the restoration of LV electrical synchrony is the key mechanism driving structural remodeling and clinical improvement with CRT, changes in QRS duration after CRT may reflect the quality of electrical resynchronization and the degree of correction of electromechanical abnormalities [10]. However, this effect is not consistently observed in all patients, highlighting variability in the electrical response to CRT. Moreover, it has been demonstrated that QRS widening is associated with suboptimal response and even with deterioration in LV function following CRT [17]. This further highlights the importance of not only considering the electrical substrate for patient selection but also assessing the electrical effects of CRT to optimize treatment evaluation.

Given the complexity of predicting response in a population with heart failure and multiple comorbidities, various strategies have been investigated, mainly based on clinical and echocardiographic criteria. If these different response criteria show poor agreement, the possibility of generalizing findings across multiple studies becomes significantly limited. Regarding echocardiographic parameters, several studies have identified a correlation between QRS shortening and concurrent changes in left ventricular end-systolic volume, a key marker of favorable reverse remodeling following CRT [9,10,16,21]. Other studies have investigated the impact of CRT on functional status, as expressed by an improvement in New York Heart Association (NYHA) functional class [19,20,22]. The lack of a standardized definition for CRT response continues to pose a significant challenge in advancing this field, as suggested by the moderate heterogeneity observed in our study. This variability reflects the absence of universally accepted response criteria in the field of CRT. Nevertheless, the consistency of our subgroup analyses supports the robustness of the association between the QRS Index and CRT response irrespective of the response definition adopted, with concordant findings observed in studies using combined clinical and echocardiographic criteria as well as those based exclusively on echocardiographic parameters.

Several baseline characteristics have been investigated and linked to an improved response to CRT, including younger age, female sex, and non-ischemic cardiomyopathy [26,27]. Additionally, some studies have suggested a role for baseline ventricular volumes, such as LVESV, with lower values being associated with a higher likelihood of clinical response [28,29,30]. Our meta-regression analysis showed that lower LVESV values were associated with even higher QRS Index values in responders compared to non-responders. This finding may indicate that patients with lower baseline LVESV have a greater probability of QRS shortening than those with higher LVESV. Moreover, our results have important prognostic implications, as it is well established that patients with lower baseline LVESV tend to exhibit more pronounced reverse remodeling and achieve better long-term outcomes following CRT [31,32].

Additionally, our findings carry clinical relevance, as QRS narrowing after CRT, assessed using the QRS Index, has shown prognostic significance. Specifically, one study identified an association with a lower incidence of all-cause mortality and cardiovascular hospitalizations [23]. The QRS Index is straightforward to calculate and can serve as a valuable tool at every stage of the CRT implantation process. During CRT implantation, the QRS Index could assist in selecting the most suitable coronary sinus branch by assessing QRS width achieved with biventricular pacing at different sites [21]. Similarly, it is worth noting that a systematic search for the site of latest electrical activation (e.g., QRS-to-Left Ventricle interval) across multiple coronary sinus branches may be time-consuming; however, electroanatomic mapping data suggest that mapping the coronary sinus itself can help identify the latest activated segment and, consequently, the branch most likely to show the greatest activation delay [33,34]. Real-world practice also highlights an implementation gap: in a recent Italian AIAC survey, most operators selected the target vein empirically, whereas only ~10% assessed electrical delay (QLV) in all coronary sinus branches; high-volume centers more frequently adopted advanced pre-procedural imaging and reported lower rates of failure in reaching the target vessel [35]. Additionally, it may help optimize the selection of the most effective pacing electrode by identifying pacing sites that achieve the greatest QRS narrowing [10]. Following the procedure, the QRS Index may help predict patients with better clinical outcomes and enable the early identification of those at risk of non-response. This could support an early CRT programming optimization or more precise titration of pharmacological therapy to enhance clinical benefits, ultimately aiming to minimize the proportion of non-responders.

Another important methodological aspect concerns the potential variability in QRS Index measurement. Differences in ECG acquisition timing after CRT implantation, device programming parameters, lead positioning, pacing configuration, and methods used for QRS duration measurement may influence the calculated QRS Index. Furthermore, variability in ECG filtering and measurement techniques across studies could introduce additional heterogeneity. Future studies should aim to standardize measurement protocols and define optimal timing for QRS Index assessment to improve reproducibility and clinical applicability.

QRS Index is a parameter that does not require expertise for its calculation, provides information at the time of implantation and does not increase costs or procedural time. While it is unlikely that a single variable can reliably predict CRT response in all eligible patients, the QRS Index holds clinical value due to its prognostic capability within a multimodal and comprehensive predictive model. By integrating multiple factors associated with response, the QRS Index could assist electrophysiologists in implementing tailored interventions to minimize the proportion of non-responders and optimize therapeutic outcomes. Future research may explore the integration of QRS Index with multimodal prediction strategies, including advanced cardiac imaging, electroanatomical mapping data, and machine learning-based prediction models. Such approaches could further improve patient selection and procedural optimization in CRT candidates.

## 5. Limitations

Our study has important limitations. First, there is the retrospective nature of most included observational studies, with the inherent risk of confounding factors. Second, there is heterogeneity in the response criteria for CRT used across studies, although our subgroup analysis based on the type of response did not reveal significant differences compared to the pooled analysis. Third, it was not possible to identify clinically meaningful threshold values for the QRS Index or to perform ROC analyses due to the lack of individual patient data across the included studies. The availability of only aggregated study-level data prevented the calculation of sensitivity, specificity, and optimal discriminatory cut-off values. Future individual patient data meta-analyses or prospective studies will be necessary to establish clinically applicable thresholds. Fourth, we included studies covering a broad time range, during which guidelines have evolved, consequently influencing patient selection. Fifth, there was a relatively short follow-up duration in the included studies, which may limit the assessment of long-term outcomes.

## 6. Conclusions

In this prognostic meta-analysis of observational studies including patients undergoing CRT, the QRS Index was significantly higher in CRT responders compared to non-responders. These findings support the QRS Index as a promising prognostic marker for predicting the likelihood of response to CRT and as a valuable tool for optimizing patient selection and early post-implantation management. Its integration into clinical decision-making may facilitate a more targeted approach, potentially reducing the proportion of non-responders and improving overall therapeutic efficacy. Further large-scale studies with extended follow-up are required to standardize this parameter in clinical practice and to evaluate its prognostic impact on major adverse cardiac events in patients receiving CRT.

## Figures and Tables

**Table 1:** Baseline characteristics of included studies.

Study	Study Design	Follow-Up (Months)	No. of Patients	Median Age (Years)	Male *n* (%)	ICM *n* (%)	AF *n* (%)	Pre LBBB *n* (%)	Pre CRT LVEF (%)	Pre CRT QRS (ms)	Risk of Bias (ROBINS-E)
**Boriani 2006 [16]**	Prospective Cohort	3	R: 13 NR: 7	62 ± 8	15 (75)	8 (40)	NA	NA	NA	155.6 ± 18.9 ms	Moderate
**Rickard 2011 [9]**	Retrospective Cohort	11.6 ± 9	R: 104 NR: 114	64.5 ± 11.8	NA	114 (52.3)	116 (53.2)	101 (46.3%)	23.7 ± 7.6	165.6 ± 26.4	Moderate
**Rickard 2012 [17]**	Retrospective Cohort	9.9	R: 72 NR: 40	69.3 ± 11.2	79 (70.5)	62 (55.4)	79 (70.5)	NA	22.5 ± 7.7	187.8 ± 23	Moderate
**Coppola** **2014 [18]**	Prospective Cohort	12	R: 26 NR: 13	NA	NA	9 (23.07)	5 (12.8)	NA	NA	NA	Moderate
**Coppola** **2016 [10]**	Prospective Cohort	12	R: 220 NR: 91	69 ± 10	225 (72)	120 (39)	18 (6)	257 (83%)	28 ± 6	166 ± 23	Moderate
**Martinez Lopez** **2019 [19]**	Retrospective Cohort	12	R: 54 NR: 37	61.2 ± 11	69 (75.8)	45 (49.5)	11 (12.1)	77 (84.6%)	25.4 ± 5.45	151.3 ± 12.6	Moderate
**Mashal** **2023 [20]**	Prospective Cohort	6	R: 40 NR: 15	58 ± 8	44 (80)	17 (30.9)	NA	NA	23 ± 5	R: 157.5 ± 22.4 NR:152.9 ± 26.9	Moderate
**Mugnai** **2023 [21]**	Retrospective Cohort	12.7 ± 4.5	R: 180 NR: 146	70 ± 10.1	250 (76.7)	137 (42)	114 (35)	213 (65.3%)	NA	NA	Moderate
**Plata-Corona 2024 [22]**	Retrospective Cohort	NA	R: 51 NR: 51	54 ± 18.7	102 (59.8)	37 (36.3)	NA	87 (85.29%)	23.7 ± 7.7	160 ± 29.4	Moderate

R: responders; NR: non-responders; ICM: ischemic cardiomyopathy; AF: Atrial Fibrillation; Pre: previous; CRT: cardiac resynchronization therapy; LBBB: left bundle branch block; NA: not available.

## Data Availability

We declare that all the raw data supporting the conclusions of this meta-analysis are available upon reasonable request.

## Supplementary Materials

The following supporting information can be downloaded at https://www.mdpi.com/article/10.3390/jcm15083074/s1: Figure S1: Bubble plot showing relationship between LVEDV and difference in QRS Index between CRT responders and non-responders. LVEDV: Left ventricular end-diastolic volume. The size of the bubbles represents the weight of each study, calculated according to a random-effects model. Blue line: Linear prediction; Figure S2: Bubble plot showing relationship between LVEF and difference in QRS Index between CRT responders and non-responders. LVEF: Left ventricular ejection fraction. The size of the bubbles represents the weight of each study, calculated according to a random-effects model. Blue line: Linear prediction; Figure S3: Bubble plot showing relationship between QRS Duration and difference in QRS Index between CRT responders and non-responders. The size of the bubbles represents the weight of each study, calculated according to a random-effects model. Blue line: Linear prediction; Figure S4: Funnel plot demonstrating a relatively symmetric distribution of studies around the pooled effect estimate, indicating a low risk of publication bias; Table S1: PRISMA Checklist; Table S2: Definitions of CRT response across the included studies. CRT: Cardiac Resynchronization Therapy; LVESV: Left Ventricular End-systolic Volume; LVEF: Left Ventricular Ejection Fraction; NYHA: New York Heart Association; RV: Right Ventricular; Table S3: Leave-one-out sensitivity analysis; Table S4: Meta-regression analysis of baseline variables and the difference in QRS Index between CRT responders and non-responders; Table S5: Quality assessment of the included studies based on the Risk Of Bias In Non-randomized Studies—of Exposures (ROBINS-E) assessment tool.

## Abbreviations

CI	Confidence Interval
CRT-D	Cardiac Resynchronization Therapy-Defibrillator
HF	Heart Failure
IQR	Interquartile Range
LVEDV	Left Ventricular End-Diastolic Volume
LVEF	Left Ventricular Ejection Fraction
LVESV	Left Ventricular End-Systolic Volume
MD	Mean Difference
NYHA	New York Heart Association
PRISMA	Preferred Reporting Items for Systematic Reviews and Meta-Analysis
ROBINS-E	Risk Of Bias In Non-randomized Studies of Exposures
SD	Standard Deviation

## References

[1] Cunnington C., Kwok C.S., Satchithananda D.K., Patwala A., Khan M.A., Zaidi A., Ahmed F.Z., Mamas M.A. (2015). Cardiac resynchronisation therapy is not associated with a reduction in mortality or heart failure hospitalisation in patients with non-left bundle branch block QRS morphology: Meta-analysis of randomised controlled trials. Heart.

[2] Cleland J.G., Abraham W.T., Linde C., Gold M.R., Young J.B., Daubert J.C., Sherfesee L., Wells G.A., Tang A.S. (2013). An individual patient meta-analysis of five randomized trials assessing the effects of cardiac resynchronization therapy on morbidity and mortality in patients with symptomatic heart failure. Eur. Heart J..

[3] Daubert J.C., Saxon L., European Heart Rhythm Association (EHRA), European Society of Cardiology (ESC), Heart Rhythm Society, Heart Failure Society of America (HFSA), American Society of Echocardiography (ASE), American Heart Association (AHA), European Association of Echocardiography (EAE) of ESC, Heart Failure Association of ESC (HFA) (2012). 2012 EHRA/HRS expert consensus statement on cardiac resynchronization therapy in heart failure: Implant and follow-up recommendations and management. Europace.

[4] Zareba W., Klein H., Cygankiewicz I., Hall W.J., McNitt S., Brown M., Cannom D., Daubert J.P., Eldar M., Gold M.R. (2011). Effectiveness of Cardiac Resynchronization Therapy by QRS Morphology in the Multicenter Automatic Defibrillator Implantation Trial-Cardiac Resynchronization Therapy (MADIT-CRT). Circulation.

[5] Migliore F., Baritussio A., Stabile G., Reggiani A., D’oNofrio A., Palmisano P., Caico S.I., De Simone A., Marini M., Pecora D. (2016). Prevalence of true left bundle branch block in current practice of cardiac resynchronization therapy implantation. J. Cardiovasc. Med..

[6] Bryant A.R., Wilton S.B., Lai M.P., Exner D.V. (2013). Association between QRS duration and outcome with cardiac resynchronization therapy: A systematic review and meta-analysis. J. Electrocardiol..

[7] Bazoukis G., Naka K.K., Alsheikh-Ali A., Tse G., Letsas K.P., Korantzopoulos P., Liu T., Yeung C., Efremidis M., Tsioufis K. (2020). Association of QRS narrowing with response to cardiac resynchronization therapy-a systematic review and meta-analysis of observational studies. Heart Fail. Rev..

[8] Ma J., Liu Y., Dong Y., Chen M., Xia L., Xu M. (2020). Association between changes in QRS width and echocardiographic responses to cardiac resynchronization therapy: A systematic review and meta-analysis. Medicine.

[9] Rickard J., Popovic Z., Verhaert D., Sraow D., Baranowski B., Martin D.O., Lindsay B.D., Varma N., Tchou P., Grimm R.A. (2011). The QRS narrowing index predicts reverse left ventricular remodeling following cardiac resynchronization therapy. Pacing Clin. Electrophysiol..

[10] Coppola G., Ciaramitaro G., Stabile G., Donofrio A., Palmisano P., Carità P., Mascioli G., Pecora D., De Simone A., Marini M. (2016). Magnitude of QRS duration reduction after biventricular pacing identifies responders to cardiac resynchronization therapy. Int. J. Cardiol..

[11] Higgins J.P.T., Thomas J., Chandler J., Cumpston M., Li T., Page M.J., Welch V.A. (2024). Cochrane Handbook for Systematic Reviews of Interventions Version 6.5.

[12] Page M.J., McKenzie J.E., Bossuyt P.M., Boutron I., Hoffmann T.C., Mulrow C.D., Shamseer L., Tetzlaff J.M., Akl E.A., Brennan S.E. (2021). The PRISMA 2020 statement: An updated guideline for reporting systematic reviews. BMJ.

[13] Higgins J.P.T., Morgan R.L., Rooney A.A., Taylor K.W., Thayer K.A., Silva R.A., Lemeris C., Akl E.A., Bateson T.F., Berkman N.D. (2024). A tool to assess risk of bias in non-randomized follow-up studies of exposure effects (ROBINS-E). Environ. Int..

[14] Wan X., Wang W., Liu J., Tong T. (2014). Estimating the sample mean and standard deviation from the sample size, median, range and/or interquartile range. BMC Med. Res. Methodol..

[15] Luo D., Wan X., Liu J., Tong T. (2018). Optimally estimating the sample mean from the sample size, median, mid-range, and/or mid-quartile range. Stat. Methods Med. Res..

[16] Boriani G., Biffi M., Martignani C., Ziacchi M., Saporito D., Grigioni F., Domenichini G., Valzania C., Diemberger I., Bertini M. (2006). Electrocardiographic remodeling during cardiac resynchronization therapy. Int. J. Cardiol..

[17] Rickard J., Jackson G., Spragg D.D., Cronin E.M., Baranowski B., Tang W.W., Wilkoff B.L., Varma N. (2012). QRS prolongation induced by cardiac resynchronization therapy correlates with deterioration in left ventricular function. Heart Rhythm.

[18] Coppola G., Bonaccorso P., Corrado E., Ciaramitaro G., Ajello L., Nugara C., Assennato P. (2014). The QRS narrowing index for easy and early identification of responder to cardiac resynchronization therapy. Int. J. Cardiol..

[19] Martínez L.F., Castañeda C.O., Falcón R.R., Hevia J.A.C., Sánchez M.D., Cardentey M.C., Betancourt A.M., López A.G., Galguera J.d.Z., Milián I.R.Q. (2019). Cardiac resynchronization therapy: QRS index as a response predictor. CorSalud.

[20] Mashal A., Soltan G., Samy N., Soliman A. (2023). Left ventricular global strain and duration of QRS complex as predictors of prognosis after cardiac resynchronization therapy. Europace.

[21] Mugnai G., Donazzan L., Tomasi L., Piccoli A., Cavedon S., Pescoller F., Bolzan B., Perrone C., Rauhe W.G., Oberhollenzer R. (2023). The usefulness of QRS Index for prediction of echocardiographic response in cardiac resynchronization therapy: A multicenter study. Minerva Cardiol. Angiol..

[22] Plata-Corona J.C., Solis-Jiménez F., Flores-Flamand M., Dattoli-García C.A., Priego-Ranero Á.A., Sierra-Lara J.D., Sierra-Fernández C.R. (2024). Response predictors to cardiac resynchronization therapy in chronic heart failure: A 10-year-cardiovascular center experience. Arch. Cardiol. Mex..

[23] Coppola G., Madaudo C., Mascioli G., D’Ardia G., La Greca C., Prezioso A., Corrado E. (2024). Tighter is better: Can a simple and cost-free parameter predict response to cardiac synchronization therapy?. Pacing Clin. Electrophysiol..

[24] Mollema S.A., Bleeker G.B., van der Wall E.E., Schalij M.J., Bax J.J. (2007). Usefulness of QRS duration to predict response to cardiac resynchronization therapy in patients with end-stage heart failure. Am. J. Cardiol..

[25] Varma N. (2009). Left ventricular conduction delays and relation to QRS configuration in patients with left ventricular dysfunction. Am. J. Cardiol..

[26] Goldenberg I., Moss A.J., Hall W.J., Foster E., Goldberger J.J., Santucci P., Shinn T., Solomon S., Steinberg J.S., Wilber D. (2011). Predictors of response to cardiac resynchronization therapy in the Multicenter Automatic Defibrillator Implantation Trial with Cardiac Resynchronization Therapy (MADIT-CRT). Circulation.

[27] Glikson M., Nielsen J.C., Kronborg M.B., Michowitz Y., Auricchio A., Barbash I.M., Barrabés J.A., Boriani G., Braunschweig F., Brignole M. (2021). 2021 ESC Guidelines on cardiac pacing and cardiac resynchronization therapy. Eur. Heart J..

[28] Rinkuniene D., Bucyte S., Ceseviciute K., Abramavicius S., Baronaite-Dudoniene K., Laukaitiene J., Kazakevicius T., Zabiela V., Sileikis V., Puodziukynas A. (2014). Predictors of positive response to cardiac resynchronization therapy. BMC Cardiovasc. Disord..

[29] Jin H., Gu M., Hua W., Fan X., Niu H., Ding L., Wang J., Xue C., Zhang S. (2017). Predictors of super-response to cardiac resynchronization therapy: The significance of heart failure medication, pre-implant left ventricular geometry and high percentage of biventricular pacing. J. Geriatr. Cardiol..

[30] Park M.Y., Altman R.K., Orencole M., Kumar P., Parks K.A., Heist K.E., Singh J.P., Picard M.H. (2012). Characteristics of responders to cardiac resynchronization therapy: The impact of echocardiographic left ventricular volume. Clin. Cardiol..

[31] Verhaert D., Grimm R.A., Puntawangkoon C., Wolski K., De S., Wilkoff B.L., Starling R.C., Tang W.H., Thomas J.D., Popović Z.B. (2010). Long-term reverse remodeling with cardiac resynchronization therapy: Results of extended echocardiographic follow-up. J. Am. Coll. Cardiol..

[32] Rao K., Rao N.S., McNitt S., Polonsky B., Goldenberg I., Solomon S., Kutyifa V. (2019). Impact of Left Ventricular End-Systolic Volume on the Effectiveness of Cardiac Resynchronization Therapy: A Madit-Crt Long-Term Sub-Study. J. Am. Coll. Cardiol..

[33] Maines M., Peruzza F., Zorzi A., Moggio P., Angheben C., Catanzariti D., Coletti M., Pangrazzi C., Del Greco M. (2020). Coronary sinus and great cardiac vein electroanatomic mapping predicts the activation delay of the coronary sinus branches. J. Cardiovasc. Electrophysiol..

[34] Vergara C., Stella S., Maines M., Africa P.C., Catanzariti D., Demattè C., Centonze M., Nobile F., Quarteroni A., Del Greco M. (2022). Computational electrophysiology of the coronary sinus branches based on electro-anatomical mapping for the prediction of the latest activated region. Med. Biol. Eng. Comput..

[35] Ziacchi M., Anselmino M., Palmisano P., Casella M., Pelargonio G., Russo V., D’oNofrio A., Massaro G., Vilotta M., Lauretti M. (2024). Selection of candidates for cardiac resynchronization therapy and implantation management: An Italian survey promoted by the Italian Association of Arrhythmology and Cardiac Pacing. J. Cardiovasc. Med..

