# Supplementary material for: QRS Index as a Predictor of Response to Cardiac Resynchronization Therapy: A Systematic Review and Meta-Analysis
Source: J Clin Med. 2026 Apr 17;15(8):3074. doi: 10.3390/jcm15083074 (PMC13116760; doi:10.3390/jcm15083074)
Supplement: Supplementary file 1 [file jcm-15-03074-s001.zip › jcm-4229705-supplementary.pdf]

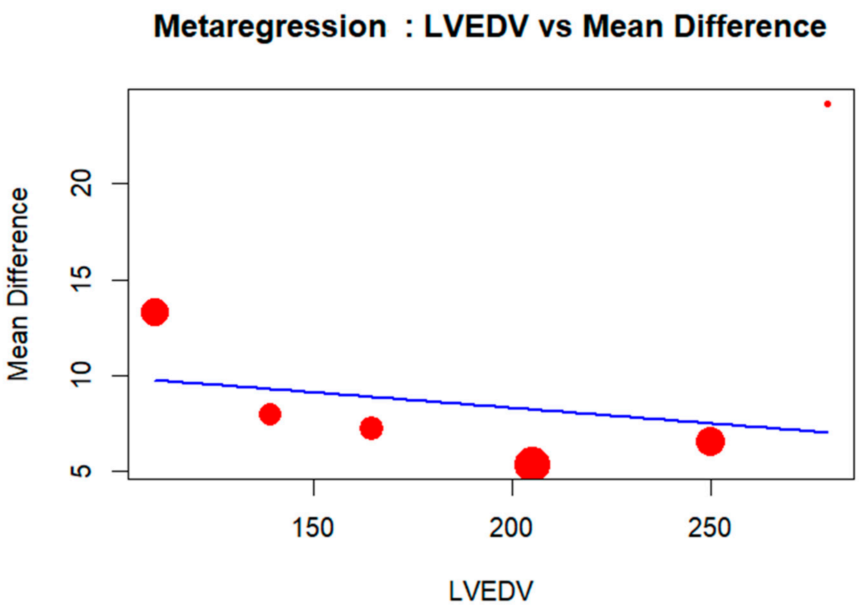

Figure S1: Bubble plot showing relationship between LVEDV and difference in QRS Index between CRT responders and non-responders. LVEDV: Left ventricular end-diastolic volume. The size of the bubbles represents the weight of each study, calculated according to a random-effects model. **Blue line:** Linear prediction

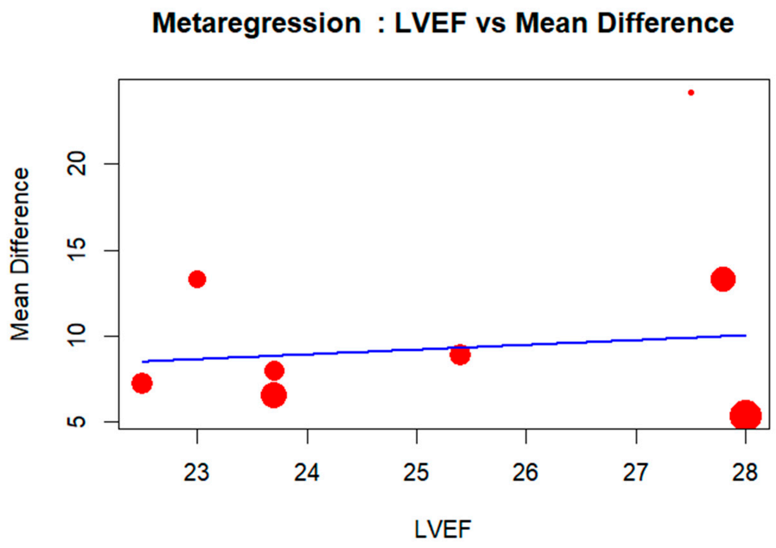

Figure S2: Bubble plot showing relationship between LVEF and difference in QRS Index between CRT responders and non-responders. LVEF: Left ventricular ejection fraction. The size of the bubbles represents the weight of each study, calculated according to a random-effects model. **Blue line:** Linear prediction.

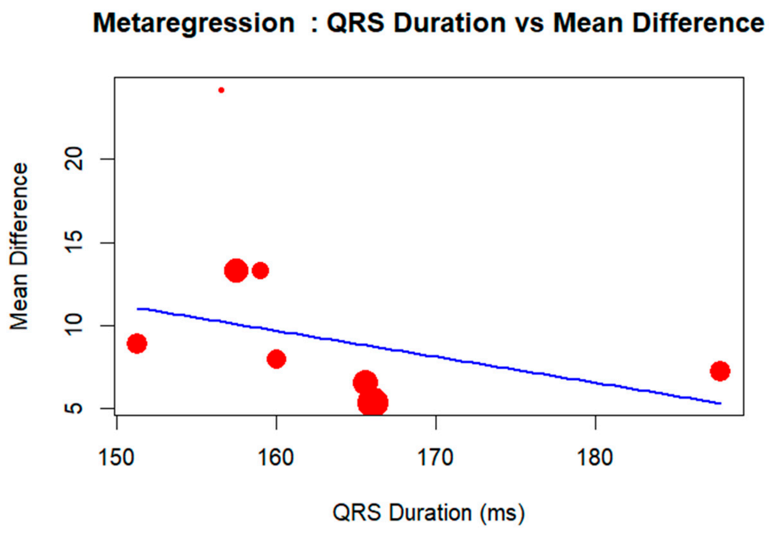

Figure S3: Bubble plot showing relationship between QRS Duration and difference in QRS Index between CRT responders and non-responders. The size of the bubbles represents the weight of each study, calculated according to a random-effects model. **Blue line:** Linear prediction.

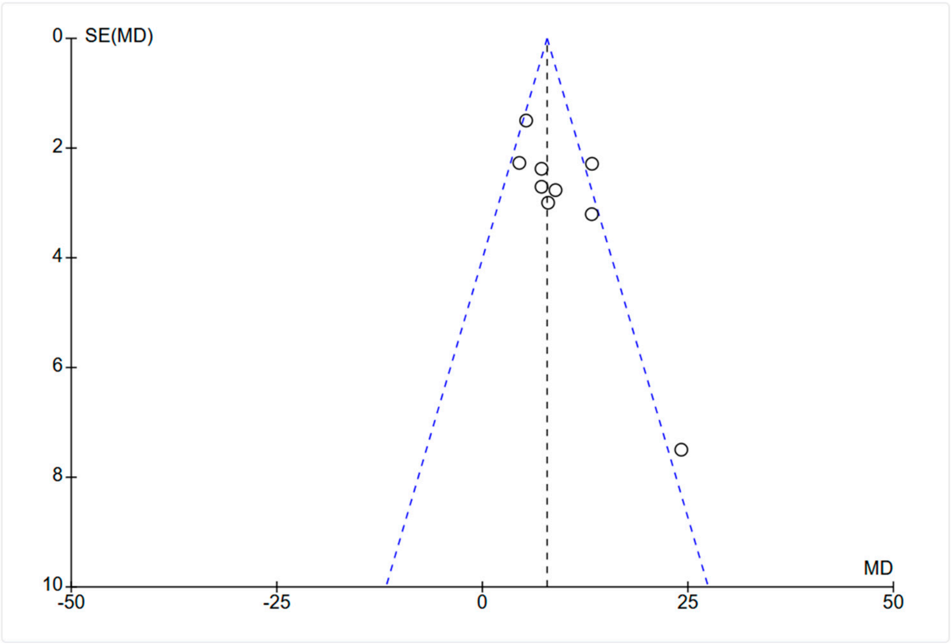

Figure S4: Funnel plot demonstrating a relatively symmetric distribution of studies around the pooled effect estimate, indicating a low risk of publication bias.

Table S1. PRISMA Checklist

Section and Topic	Item #	Checklist item	Location where item is reported
**TITLE**			
Title	1	Identify the report as a systematic review.	Title
**ABSTRACT**			
Abstract	2	See the PRISMA 2020 for Abstracts checklist.	Abstract section
**INTRODUCTION**			
Rationale	3	Describe the rationale for the review in the context of existing knowledge.	Introduction paragraphs 1-3
Objectives	4	Provide an explicit statement of the objective(s) or question(s) the review addresses.	Last paragraph of Introduction
**METHODS**			
Eligibility criteria	5	Specify the inclusion and exclusion criteria for the review and how studies were grouped for the syntheses.	Methods – eligibility criteria paragraph
Information sources	6	Specify all databases, registers, websites, organisations, reference lists and other sources searched or consulted to identify studies. Specify the date when each source was last searched or consulted.	Methods – Search strategy
Search strategy	7	Present the full search strategies for all databases, registers and websites, including any filters and limits used.	Methods – Search strategy
Selection process	8	Specify the methods used to decide whether a study met the inclusion criteria of the review, including how many reviewers screened each record and each report retrieved, whether they worked independently, and if applicable, details of automation tools used in the process.	Methods – Search strategy and data extraction
Data collection process	9	Specify the methods used to collect data from reports, including how many reviewers collected data from each report, whether they worked independently, any processes for obtaining or confirming data from study investigators, and if applicable, details of automation tools used in the process.	Methods – Search strategy and data extraction
Data items	10a	List and define all outcomes for which data were sought. Specify whether all results that were compatible with each outcome domain in each study were sought (e.g. for all measures, time points, analyses), and if not, the methods used to decide which results to collect.	Methods – primary endpoint paragraph
	10b	List and define all other variables for which data were sought (e.g. participant and intervention characteristics, funding sources). Describe any assumptions made about any missing or unclear information.	Methods – meta-regression variables
Study risk of bias assessment	11	Specify the methods used to assess risk of bias in the included studies, including details of the tool(s) used, how many reviewers assessed each study and whether they worked independently, and if applicable, details of automation tools used in the process.	Methods – Quality assessment
Effect measures	12	Specify for each outcome the effect measure(s) (e.g. risk ratio, mean difference) used in the synthesis or presentation of results.	Methods – Statistical analysis
Synthesis methods	13a	Describe the processes used to decide which studies were eligible for each synthesis (e.g. tabulating the study intervention characteristics and comparing against the planned groups for each synthesis (item #5)).	Methods – eligibility criteria + pooled analysis description
	13b	Describe any methods required to prepare the data for presentation or synthesis, such as handling of missing summary statistics, or data conversions.	Methods – Statistical analysis
	13c	Describe any methods used to tabulate or visually display results of individual studies and syntheses.	Methods – forest

Study	Clinical criteria	Echocardiographic criteria	Combined	Definition used
**Boriani 2006**	Not used	**Yes**	No	**>15% reduction in LVESV** after 3 months of BiV pacing.
**Coppola 2014**	Not used	**Yes**	No	**>10% reduction in LVESV** at 6 months.
**Coppola 2016**	Not used	**Yes**	No	**10% reduction in LVESV** at 6 months.
**Martínez-López 2019**	**Yes**	**Yes**	**Yes**	Fulfillment of all of the following: **improvement in NYHA functional class, 5% increase in LVEF, and 10% reduction in LVESV**.
**Mashal 2023**	**Yes**	**Yes**	**Yes**	**Improvement in NYHA functional class by at least one class and either ≥15% reduction in LVESV or >10% increase in LVEF**.
**Mugnai 2023**	Not used	**Yes**	No	**≥15% reduction in LVESV or ≥5% increase in LVEF**.
**Plata-Corona 2024**	**Yes**	**Yes**	**Yes**	**Improvement in NYHA functional class by at least one category associated with ≥5% increase in LVEF**.
**Rickard 2011**	Not used	**Yes**	No	**≥10% reduction in LVESV** from baseline.
**Rickard 2013**	Not used	**Yes**	No	**≥15% reduction in LVESV** from baseline.

Table S2. Definitions of CRT response across the included studies. CRT: Cardiac Resynchronization Therapy; LVESV: Left Ventricular End-systolic Volume; LVEF: Left Ventricular Ejection Fraction; NYHA: New York Heart Association; RV: Right Ventricular.

Table S3. Leave-one-out sensitivity analysis.

Excluded study	QRS Index in responders vs non-responders		
	Mean difference (95% CI)	Heterogeneity	
		I^2, %	*P* value
None	8.76 (6.45–11.06)	45	<0.00001
Boriani 2006	8.36 (6.15–10.58)	43	<0.00001
Coppola 2014	9.09 (6.43–11.74)	52	<0.00001
Coppola 2016	9.46 (7.14-11.77)	29	<0.00001
Martinez Lopez 2019	8.84 (6.22–11.45)	52	<0.00001
Mashal 2023	8.25 (5.97–10.53)	41	<0.00001
Mugnai 2023	7.61 (5.70–9.52)	14	<0.00001
Plata-Corona 2024	8.93 (6.34–11.53)	53	<0.00001
Rickard 2011	9.18 (6.56–11.81)	50	<0.00001
Rickard 2013	9.05 (6.43–11.67)	52	<0.00001

**Table S4.** Meta-regression analysis of baseline variables and the difference in QRS Index between CRT responders and non-responders.	**Number of studies**	**β**	**95% CI**	**P**
LVESV	7	-0.0483	-0.0938; -0.0029	0.0372
LVEDV	6	-0.0161	-0.0760; 0.0437	0.5974
LVEF	8	0.2777	-1.0936; 1.6490	0.6914
QRS Duration	8	-0.1570	-0.4093; 0.0952	0.2224

**Study**	**Bias due to confoundi ng**	**Bias arising from measure ment of the exposure**	**Bias in selection of participan ts**	**Bias due to post-exposure interventi ons**	**Bias due to missing data**	**Bias in measure ment of outcomes**	**Bias in selection of the reported result**	**Overall risk of bias judgemen t**
**Boriani 2006**	Moderate	Low	Low	Low	Low	Moderate	Low	Moderate
**Coppola 2014**	Moderate	Low	Low	Moderate	Low	Low	Moderate	Moderate
**Coppola 2016**	Moderate	Low	Low	Moderate	Moderate	Low	Low	Moderate

**Martinez Lopez 2019**	Moderate	Low	Moderate	Low	Moderate	Moderate	Low	Moderate
**Mashal 2023**	Moderate	Low	Low	Low	Low	Low	Moderate	Moderate
**Mugnai 2023**	Moderate	Low	Low	Moderate	Low	Low	Moderate	Moderate
**Plata Corona 2024**	Moderate	Low	Moderate	Moderate	Low	Low	Low	Moderate
**Rickard 2011**	Moderate	Low	Low	Low	Low	Moderate	Moderate	Moderate
**Rickard 2013**	Moderate	Low	Moderate	Moderate	Low	Low	Moderate	Moderate

LVESV: Left ventricular end-systolic volumen; LVEDV: Left ventricular end-diastolic volumen; LVEF: Left ventricular ejection fraction.

Table S5. Quality assessment of the included studies based on the Risk Of Bias In Non-randomized Studies – of Exposures (ROBINS-E) assessment tool.
